# The Snacking Chameleon: Psychological Proximity Increases Imitation of Food Intake Independently of Brand Choice

**DOI:** 10.3390/foods9020228

**Published:** 2020-02-21

**Authors:** Claudia Bischoff, Leonie Reutner, Jochim Hansen

**Affiliations:** 1 Department of Psychology, University of Salzburg, 5020 Salzburg, Austria; 2 Department of Pharmaceutical Sciences, University of Basel, 4056 Basel, Switzerland; leonie.reutner@unibas.ch

**Keywords:** imitation, social modeling, psychological distance, food intake, snacking behavior

## Abstract

Observing other people snacking can affect one’s own consumption behavior. The present experiment tested whether temporal distance moderates imitation of brand choice and the number of snacks consumed. Based on previous research demonstrating that psychological distance (e.g., temporal or spatial distance) reduces imitation of movements, we hypothesized that participants would imitate the amount of food intake to a lesser degree when they temporally distance themselves from a model person. To test this idea, participants (*n* = 113) were asked to imagine their life either the next day (proximal condition) or in one year (distant condition). Next, participants watched a video clip depicting a model person who chose one of two brands of pretzels and ate either plenty or just a few of the pretzels. Then, participants chose one of the two brands of pretzels, served themselves as many of the pretzels as they liked, and ate them while filling in a tasting questionnaire. As expected, participants primed with proximity imitated snack intake more than participants primed with distance. The brand choice was not affected by self-distancing. Implications for snacking behavior are discussed.

## 1. Introduction

A person’s food intake is affected by the food intake of other people: previous research consistently found that one is likely to eat more when one’s companion eats a large amount, and to eat less when one’s companion eats a small amount (for a review, see [1]). Such social modeling of food intake has been shown for different populations and eating contexts [2,3,4,5,6,7]. The effect seems to be relatively independent of one’s hunger or satiety level [8]. Additionally, social modeling of food intake has been found for different types of food, such as ice cream (e.g., [9,10]), cookies (e.g., [11,12]), pizza (e.g., [7]), or fruits and vegetables (e.g., [7]). The present study investigated whether this effect is moderated by psychological distance. Specifically, whether temporal distance reduces the influence of observing others’ snacking behavior on one’s own snacking behavior was tested.

One explanation of social modeling effects of food consumption is that one may monitor others’ eating behavior in order to reduce one’s own uncertainty about how much to eat [13]. In other words, people may use the food intake of an observed model as a norm for their own food intake. Additionally, and independently of social norms, automatic imitation of movements may explain the influence of observing others’ consumption on one’s own consumption [14]. For instance, it has been shown that young adults imitate the drinking behavior of peers or movie actors by taking a sip directly after the observed person has taken a sip [15,16]. Likewise, observing another person eating a snack directly triggers a similar reaction in the observer [14], indicating that imitation of concrete movements may contribute to social modeling of food intake. Given that social modeling of food intake is based—at least in part—on imitation of movements, psychological distance may moderate the effect because psychological distance has been shown to reduce imitation of movements.

Individuals reproduce near behavior, compared with distant behavior, in a more literal action-by-action manner [17]. In one study, for instance, New York University students were asked to learn a new activity by watching a video of a model performing the activity ([17] Exp. 2). Spatial distance from the model was manipulated by telling half of the participants that the video was made in a spatially proximal location (i.e., New York) and telling the other half of the participants that the video was made in a spatially distant location (i.e., Los Angeles). The analysis of participants’ imitation of the activity showed that the model’s movements were more likely to be imitated when participants believed the model was near (New York) rather than distant (Los Angeles). Likewise, people are more likely to imitate gestures when they think about the near future compared to when they think about the distant future [18].

These effects of distance on imitation can be explained by construal level theory [19,20,21], which proposes that psychological distance (e.g., spatial, temporal, or social distance) increases the abstractness of mental representation. Any action can be represented at different levels of construal [22,23,24,25]. Lower-level construals are concrete, relatively unstructured, and contextualized representations that include subordinate and incidental features of events. Higher-level construals are abstract, schematic, and decontextualized representations that extract the gist from the available information. They emphasize superordinate, core features of events, omitting specific features that may vary without significantly changing the meaning of events. For example, representing the action of drinking coffee as “enjoying oneself” as opposed to “moving the cup to the lips” omits its information about the movements and objects involved in the actions (e.g., the cup, the lips, and the arms) but communicates its core meaning and conveys a more general understanding of the behavior. Such abstract, high-level construals usually refer to the superordinate purpose of the behavior (by indicating why it is performed) rather than to its subordinate means (how it is performed).

When an action is psychologically distant (e.g., in time or space), individuals represent the action at a higher level, usually in terms of its goal. Near actions, in contrast, are represented relatively more in terms of their specific means and movements. Accordingly, research has shown that framing the same action as near rather than distant leads participants to represent it in terms of concrete, more incidental, and potentially changeable means rather than in terms of higher-level goals that convey its relatively invariant essence [19,26]. The more concrete representation of near actions may explain why individuals are more likely to imitate movements when they believe the behavior to be spatially or temporally close than when they believe the behavior to be spatially or temporally distant.

Consistent with this view, it has been demonstrated that abstract features of behavior (such as goals), in contrast, are less likely to be imitated when psychological distance is low than when distance is high [27,28]. In one study, for instance, participants watched a model pressing one of two keys on the keyboard (i.e., the goal of the action) with either the left or right hand across several trials (i.e., the movement [27]). When participants were asked to imitate the observed behavior, they made more errors in pressing the correct key—that is, they less likely imitated the goal—if the observed behavior had been presented spatially near, compared with spatially distant. This finding indicates that individuals are better able to focus on goals when imitating distant (compared to proximal) actions.

The present study was designed to test whether temporal distance reduces imitation of food intake, which has not been tested so far. In particular, we investigated whether mental distancing from the current situation by imagining oneself in a distant (vs. near) future would affect the imitation of food intake. We hypothesized that mentally “zooming out” of a situation has comparable effects of imitation as the actual distance from a model. Moreover, we tested the hypothesis that imitation of concrete eating behavior (i.e., imitation of the number of snacks eaten) will be reduced by the temporal distance but not imitation of abstract, contextually more invariant features of the situation. That is, we did not expect imitation of the choice of snack brand to be affected by psychological distance.

## 2. Method

### 2.1. Participants and design

One hundred and eighteen students of various majors participated in the laboratory study at the University of Salzburg in exchange for candy bars and course credit. Participants were recruited by a note that was placed at the notice-board of the University of Salzburg. Students that were interested in joining the study could contact us via email. In addition, we asked people in the hallways if they would like to join a study. Participants were randomly assigned to a 2 (temporal distance: distant vs. proximal) × 2 (quantity consumed by model: small vs. large) × 2 (brand chosen by model: brand one vs. brand two) between-participants design. We determined the sample size based on the effect size found in related research (i.e., η_p_^2^ = 0.06, see [27] p. 1036), which translates to *f* ≈ 0.26. Entering this effect size and a desired power of 1–β = 0.80, an alpha level of α = 0.05 into the program G*Power [29] with specifying the numerator *df* = 1 and number of groups = 4 resulted in a total sample size of 119. We based our power analysis on four groups because we were interested in the 2 × 2 interactions and not the full 2 × 2 × 2 interaction. Unfortunately, we had to exclude five participants from the collected sample of 118 participants because the experimenter accidentally administered the consumption task prior to showing the video. The remaining sample consisted of 113 participants (86 females, 27 males). Age ranged from 15 to 62 years (*M* = 24,2, *SD* = 6.89, *Mdn* = 22).

### 2.2. Materials and Procedure

The experiment was conducted in the laboratory and was carried out in accordance with the Code of Ethics of the World Medical Association (Declaration of Helsinki) and the institutional ethics guidelines of the University of Salzburg (Statutes of the University of Salzburg §163). All participants consented to participate on a voluntary basis by signing a written consent form.

**Figure 1:**
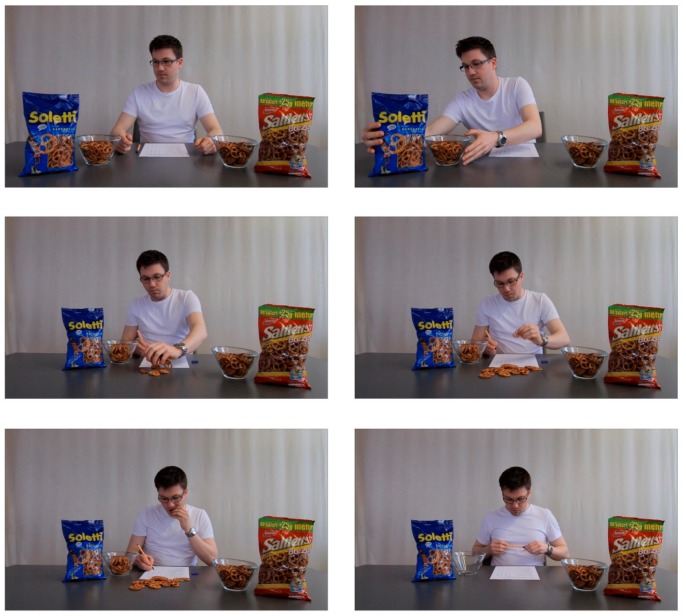
Screenshots of one of the videos shown to participants (large quantity of the brand “Soletti” was eaten by the model).

After participants entered the laboratory, they were welcomed and seated in a cubicle where they were given the opportunity to drink a glass of apple juice in order to ensure similar thirst levels across participants since thirst could have possibly influenced the consumption of the pretzels. Next, temporal self-distancing was manipulated: participants were asked to write 5 to 10 sentences either about their life on the next day (temporal proximity) or about their life on a day in one year (temporal distance). Specifically, they were instructed to “imagine your typical daily routine tomorrow [in one year] and what you might feel, think, or do” and then to write their freely occurring thoughts in a space provided. Such a self-distancing manipulation had been successfully used in previous research (e.g., [30,31,32]). After that, the experimenter told participants that they could take part in a pretzel tasting. They learned that they would watch a video where they could see what a professional tasting usually looks like. We decided to use a video model instead of a real confederate (as has been done in previous studies, e.g., [2,3,13,33]) in order to reduce the effect of social motives in the eating situation. It has been shown that people conform to others’ eating behavior to reach social acceptance or approval [3,5,13,34]. With the usage of a video model, such effects are minimized because the model person obviously could not observe or judge our participants. Participants watched a video, in which a model chose one of two brands of pretzels (i.e., Soletti or Saltletts, depending on the assigned condition). We selected these two brands because they were the leaders in the local market and had about the same quality [35]. Depending on the assigned condition, the model in the video ate either a small or a large number of pretzels while filling in a questionnaire (see Figure 1). After watching the video, participants were asked to choose between the same two pretzel brands as the model and eat as much as they like. Specifically, we placed two bowls each containing 113 g of pretzels in front of a package of each brand. Participants selected one out of the two bowls, as in the tasting video. During consumption, participants filled in a bogus paper-pencil questionnaire with nine questions about the pretzels (e.g., taste, look, texture, and wrapping). These questions had only the goal to give participants some time to taste the pretzels. The self-distancing manipulation had no effect on these measures, all *t*s < 1.23 and *Χ*^2^ < 1 (for item #7), all *p*s > 0.223. At the end of the study, participants indicated when they had eaten their last meal, how hungry and thirsty they were before the study (on 6-point scales), and provided demographic data (i.e., sex, age, nationality, mother tongue, profession, and semester). Finally, participants were debriefed, thanked, and rewarded with candy bars and course credit.

Before participants started with the session, the experimenter weighed the bowl of pretzels. After participants had finished the session, the experimenter weighed the bowl of pretzels again and calculated the difference between the two weights in order to measure how much participants had eaten. Stimulus material and data of the experiment are available at the Open Science Framework (OSF, [36]). 

## 3. Results

To test our hypothesis that participants primed with temporal proximity would more likely imitate the model with regard to the quantity he consumed compared to participants primed with temporal distance, we conducted a 2 (temporal distance: distant vs. near) × 2 (consumed quantity by model: high vs. low) analysis of variance. The alpha level was set at 0.05. Our dependent variable was the amount of pretzels in grams that participants ate.

**Figure 2:**
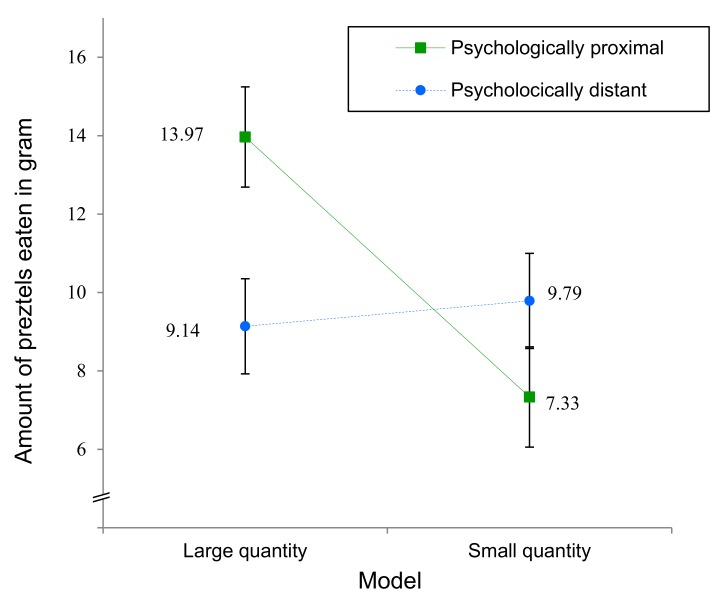
Amount of pretzels eaten as a function of psychological distance and quantity eaten by the model. Error bars indicate +/– 1 *SE*.

We found a main effect of the quantity eaten by the model on the amount of pretzels eaten by the participants, *F*(1, 109) = 4.029, *p* = 0.047, η_p_^2^ = 0.036. Specifically, participants ate more pretzels when the model ate a lot (*M* = 11.55 g, *SD* = 8.74) and fewer pretzels when the model ate only few pretzels (*M* = 8.58 g, *SD* = 7.34). As expected, this main effect was qualified by a significant interaction between self-distancing and the model’s consumed quantity, showing that participants primed with proximity more strongly imitated the model than participants primed with distance, *F*(1, 109) = 5.962, *p* = 0.016, η_p_^2^ = 0.052 (see Figure 2). Specifically, participants primed with proximity consumed fewer pretzels when they saw the model consuming a small amount of pretzels (*M* = 7.33 g, *SD* = 6.64) than when they saw the model consuming a large amount of pretzels (*M* = 13.97 g, *SD* = 10.03), *F*(1, 109) = 9.805, *p* = 0.002, η_p_^2^ = 0.083 for the contrast. Participants primed with distance, in contrast, did not show this effect (*M*_small amount_ = 9.79 g, *SD* = 7.88 vs. *M*_large amount_ = 9.14 g, *SD* = 6.53), *F* < 1 for the contrast. These findings support our hypothesis that individuals are more likely to imitate food intake when they observe consumption behavior and self-distancing is low (vs. high). The main effect of psychological distance was not significant, *F* < 1. Including the brand chosen by model as factor in this analysis only slightly alters the found effects: *F*(1, 105) = 3.416, *p* = 0.067, η_p_^2^ = 0.032 for the main effect of psychological distance and *F*(1, 105) = 6.469, *p* = 0.012, η_p_^2^ = 0.058 for the interaction between psychological distance and quantity consumed by model. There was no main effect of the brand chosen by model, *F*(1, 105) = 2.347, *p* = 0.129, η_p_^2^ = 0.022, and the brand chosen by model did not interact with the other factors, all *F*s < 1.

For brand choice, we conducted a Chi-Square test with the imitation of the model’s brand choice as the dependent variable and self-distancing as a between-subject factor. The results show that brand choice was equally often imitated independently of the distance prime, χ^2^ < 1. About half of the participants imitated the brand choice of the model (*n* = 55 of 113). This ratio was the same, independently of whether participants were primed with temporal distance (*n* = 28 of 57) or proximity (*n* = 27 of 56). This finding indicates that self-distancing may not affect imitation of brand choice.

## 4. Discussion

The present study sheds light on conditions under which observing other people snacking can affect one’s own consumption behavior. As we know from previous research [2,3,4,13,14], observing others who are snacking a lot or who are restraining themselves can lead to the same snacking behavior (i.e., eating a lot or eating less). However, there are contextual factors that appear to modify the modeling effect of imitation on snacking behavior. Current theories of norms are not able to explain why some models are more influential than others. Former research relates this fact to the social identity approach, which proposes that social influence essentially emerges from those perceived to be fellow in-group members [37]. Therefore, if a model is perceived as socially close, it enhances imitation on eating behavior because individuals regard norms set by in-group members relevant to the self, compared to out-group members [38].

Our research adds to this by proposing that distancing in general would moderate social modeling of food intake. Specifically, the present study tested the moderating effect of temporal self-distancing. Based on construal level theory [20,21], we investigated whether mental distancing from the current situation by imagining oneself in a distant (vs. near) future would affect the imitation of food intake. We hypothesized that imitation of a concrete eating behavior will be reduced by the temporal distance (i.e., imitation of the number of snacks eaten), but the imitation of abstract, contextually more invariant features of the situation (i.e., imitation of the brand of snacks eaten) will not. In our study, participants primed with proximity imitated the model’s consumed quantity, whereas participants primed with distance did not. In other words, if the model ate a lot, participants primed with proximity also ate a lot, whereas if the model ate less, participants primed with proximity also ate less, compared to participants primed with distance. This finding adds to previous research showing that imitation of food intake is weaker for socially distant models [7,38,39]. Additionally, some studies that used a video model, who was shown in a spatially distant and different environment, did not find any evidence of imitation ([40] Exp. 2 and 3; see also [38]). The present study demonstrates that temporal self-distancing has a comparable effect and supports the idea that psychological distance in general (e.g., social, spatial, and temporal distance) may reduce imitation of food intake.

The choice of the brand was not affected by self-distancing. This absence of an effect of distancing on brand choice could be due to the fact that a brand of a snack is a more stable and invariant feature than the amount consumed. However, we have to be very careful with interpreting this null effect because it might also be the case that any effect of distancing on brand choice is very subtle so our study might not have had enough power to detect it. Furthermore, a ceiling effect might have obscured any influence of self-distancing on brand choice, since most participants chose Saltletts. One could speculate that Saltletts might be better known among the participants and therefore be the preferred brand—even though we had taken great care to select two comparable brands—but we do not have any data on this conjecture. Another limitation of our findings is that we only tested salty snacks. It would be desirable to replicate this finding with other food, such as sweet snacks or healthy food. However, modeling effects of food intake have been shown to be relatively low with healthy food [8,41]. It may be interesting to see if introducing temporal or social proximity between the self and the situation may be able to increase modeling effects, even with healthy food.

The findings of the present study have practical implications for marketing purposes. On the one hand, marketers could (ab)use this effect to cause consumers to buy and consume more than they normally would. Based on construal level theory [20,21], one can assume that self-distancing from a situation would be low if an advertisement is created in a concrete way so that it appears psychologically close to the recipient. For instance, an advertisement would be perceived as concrete and close to the recipient by showing the same social group of people [42,43], at the same location [17,26], or the same time [18,19,20]. This would cause a concrete construal of the ad contents, and possible imitation of the shown behavior would focus on concrete situational aspects, such as the amount eaten by the model person. On the other hand, the found effect could be used to provide a strategy to reduce imitation. if a person keeps an abstract mindset by self-distancing herself or himself from the situation, imitation and possibly overeating in the face of a hungry companion may be reduced. This may help to stay with a diet plan that would not be affected by the current situation.

In sum, the present research demonstrates a situational factor that moderates social modeling of food intake. Temporal self-distancing reduced imitation of food intake, compared to temporal proximity.
